# Uncertainty quantification based cloud parameterization sensitivity analysis in the NCAR community atmosphere model

**DOI:** 10.1038/s41598-020-74441-x

**Published:** 2020-10-15

**Authors:** Raju Pathak, Sandeep Sahany, Saroj K. Mishra

**Affiliations:** 1 Centre for Atmospheric Sciences, Indian Institute of Technology Delhi, Hauz Khas, New Delhi, India; 2 Centre for Climate Research, Singapore, Singapore

**Keywords:** Climate sciences, Atmospheric science, Climate change

## Abstract

Using uncertainty quantification techniques, we carry out a sensitivity analysis of a large number (17) of parameters used in the NCAR CAM5 cloud parameterization schemes. The LLNL PSUADE software is used to identify the most sensitive parameters by performing sensitivity analysis. Using Morris One-At-a-Time (MOAT) method, we find that the simulations of global annual mean total precipitation, convective, large-scale precipitation, cloud fractions (total, low, mid, and high), shortwave cloud forcing, longwave cloud forcing, sensible heat flux, and latent heat flux are very sensitive to the threshold-relative-humidity-for-stratiform-low-clouds ($$rhminl)$$ and the auto-conversion-size-threshold-for-ice-to-snow $$\left( {dcs} \right).$$ The seasonal and regime specific dependence of some parameters in the simulation of precipitation is also found for the global monsoons and storm track regions. Through sensitivity analysis, we find that the Somali jet strength and the tropical easterly jet associated with the south Asian summer monsoon (SASM) show a systematic dependence on $$dcs$$ and $$rhminl$$. The timing of the withdrawal of SASM over India shows a monotonic increase (delayed withdrawal) with an increase in $$dcs$$. Overall, we find that $$rhminl$$, $$dcs$$, $$ai,$$ and $$as$$ are the most sensitive cloud parameters and thus are of high priority in the model tuning process, in order to reduce uncertainty in the simulation of past, present, and future climate.

## Introduction

Reliable projections of future climate change (CC) are of utmost importance for the assessment of impacts and adaptation planning. Quantifying and reducing uncertainties in climate projections are of high priority. In general, ensemble simulations with multiple global climate models (GCMs) or a single model with different parameterizations have been used in the past to characterize uncertainties due to physical and/or dynamical processes^1–4^. Insufficient physical understanding, coarse grid resolution, and the resolution dependency of parameterization schemes have limited the ability of GCMs to simulate CC reliably^4–8^. In GCMs, subgrid-scale processes are parameterized using some physical assumptions and empirically designed parameters. The empirically designed parameters lead to various levels of uncertainties in model simulations^9,10^.

The process of parameter calibration (or tuning) adjusts the parameter values to better match the simulated climate features with those observed. Parameter values are typically determined from limited measurements (or from theoretical calculations). Tuning becomes more cumbersome when the number of uncertain parameters is too large^11^. In this situation, researchers use an alternative statistical approach (known as a sensitivity analysis (SA)) to screen out the most sensitive parameters for calibrating GCMs. SA reduces the required number of iterations for parameter tuning, and the computational cost, without affecting the model performance^12–15^. SA is broadly categorized into two types-local and global, and it can adopt either a qualitative or quantitative approach^16–23^. The main difference between local SA (LSA) and global SA (GSA) is that the LSA approach explores the changes of model performance by perturbing one parameter at a time while keeping all other parameters fixed, whereas the GSA approach explores the model performance by perturbing all parameters at the same time. The simplest and most preferred LSA is the differential LSA, which uses partial derivatives at a fixed parameter location as the measure of parametric sensitivity. On the other hand, GSA involves two steps: (a) generating a sample set of parameters within their feasible range using a particular sampling method (e.g., Monte Carlo, Latin Hypercube^24^ (LH), Orthogonal Array^25^ (OA), and OA based LH^26^), and (b) computing the qualitative or quantitative attribution of variation in the simulation of a climate variable to the perturbation in parameter values. Some GSA methods require special sampling techniques. For example, Morris One-At-a-Time (MOAT) uses the Morris sampling technique^17^, and the Sobol method uses the Saltelli sampling technique^13^.

However, most of the discussed sampling methods require more than 10,000 simulations to cover the full parametric space and increase exponentially with an increase in the number of uncertain parameters, thus, requiring more computational resources. Therefore, researchers use an alternative cost-effective emulator to estimate the model outputs using a small number of model simulations from a specific sampling technique at the selected points, to minimize the high computing resource demand^19,27–36^. In this study, we perform the qualitative SA analysis using the Morris method^37,38^ due to its computational efficiency. This method is quite efficient in determining the few potentially important ones amongst a large number of selected parameters. However, a drawback of the Morris approach is that it cannot distinguish the non-linear effects of a parameter from the interaction effects between different parameters, and cannot estimate the effect of a parameter in relation to other parameters^37^.

The effect of deep convection on climate sensitivity has been widely studied for global as well as regional climate simulations by many researchers^6,37–40^. The effect of cloud microphysics and macrophysics has also been studied extensively, and its representation in global and regional climate models has been substantially improved^23,38,39,41^. However, it remains the dominant source of large uncertainty in climate simulations^28,31,35,42–44^. Clouds are centrally important in climate studies to understand the radiative energy budget of the Earth’s atmosphere system, hydrological cycle, and precipitation. For example, low-level clouds play a crucial role in the radiation budget of the Earth by modulating the shortwave cloud radiative forcing, and high clouds play a vital role in the radiation budget by modulating the longwave cloud radiative forcing. However, their proper representation in GCMs has been an unresolved issue^45–47^. Recently, Zelinka et al.^48^ using CMIP6 models have shown an increase in climate sensitivity due to a weaker response of extratropical cloud cover and water content to a change in surface temperature, and such change is noticed to be linked to cloud parameterizations.

Some of the above studies have carried out SA to quantify uncertainties in the simulation of few global climate variables to cloud-related parameter perturbation but limited only to few parameters. Qian et al.^23^ have reported that global mean precipitation does not respond linearly and monotonically to the change in some cloud parameters. He et al.^39^ have shown that increasing the value of ice- and snow-fall speed parameters can lower the value of longwave cloud forcing (LWCF), and a larger value of auto-conversion size threshold for ice to snow ($$dcs)$$ can lead to larger LWCF. Sanderson et al.^49^ have reported that the uncertainty in ice-fall speed can significantly affect climate sensitivity. Zhang et al.^37^ have reported that changes in threshold relative humidity parameter for high clouds can substantially affect the model performance, and reducing its value can increase the stratiform condensation and decrease atmospheric humidity. Golaz et al.^27^ have reported that the cloud droplet number limiter ($$cdnl$$) has the largest source of uncertainty in cloud-aerosol interaction. In addition, Bony and Dufresne^50^ have reported spatially varying cloud feedback and noted the trade cumulus regime to be most important for cloud feedback. Hazra et al.^51^ have reported a significant bias in the simulation of the meridional tropospheric temperature gradient and vertical moisture distribution due to improper vertical cloud distribution over south Asia, and hence weaker Indian summer monsoon simulation^52,53^.

It is, therefore, crucial to systematically characterize the sensitivity of climate simulations at global and regional scales during different seasons (especially for different monsoon and storm track regions), as well as the characteristics of South Asian summer monsoon (SASM) to cloud microphysics and macrophysics parameters to reduce uncertainty in climate simulations. The quantification and reduction of uncertainty in climate simulations due to parameter sensitivity over the SASM region are also one of the main objectives of the DST CoE in Climate Modeling, IIT Delhi, India, for the improvement of SASM simulations^5–8,54^. In this paper, we examine the GSA for the majority of physical parameters in microphysics and macrophysics parameterizations used in the NCAR CAM5, using MOAT sensitivity analysis for different simulated climate variables.

## Results and discussion

### Global spatial variance distribution

**Figure 1:**
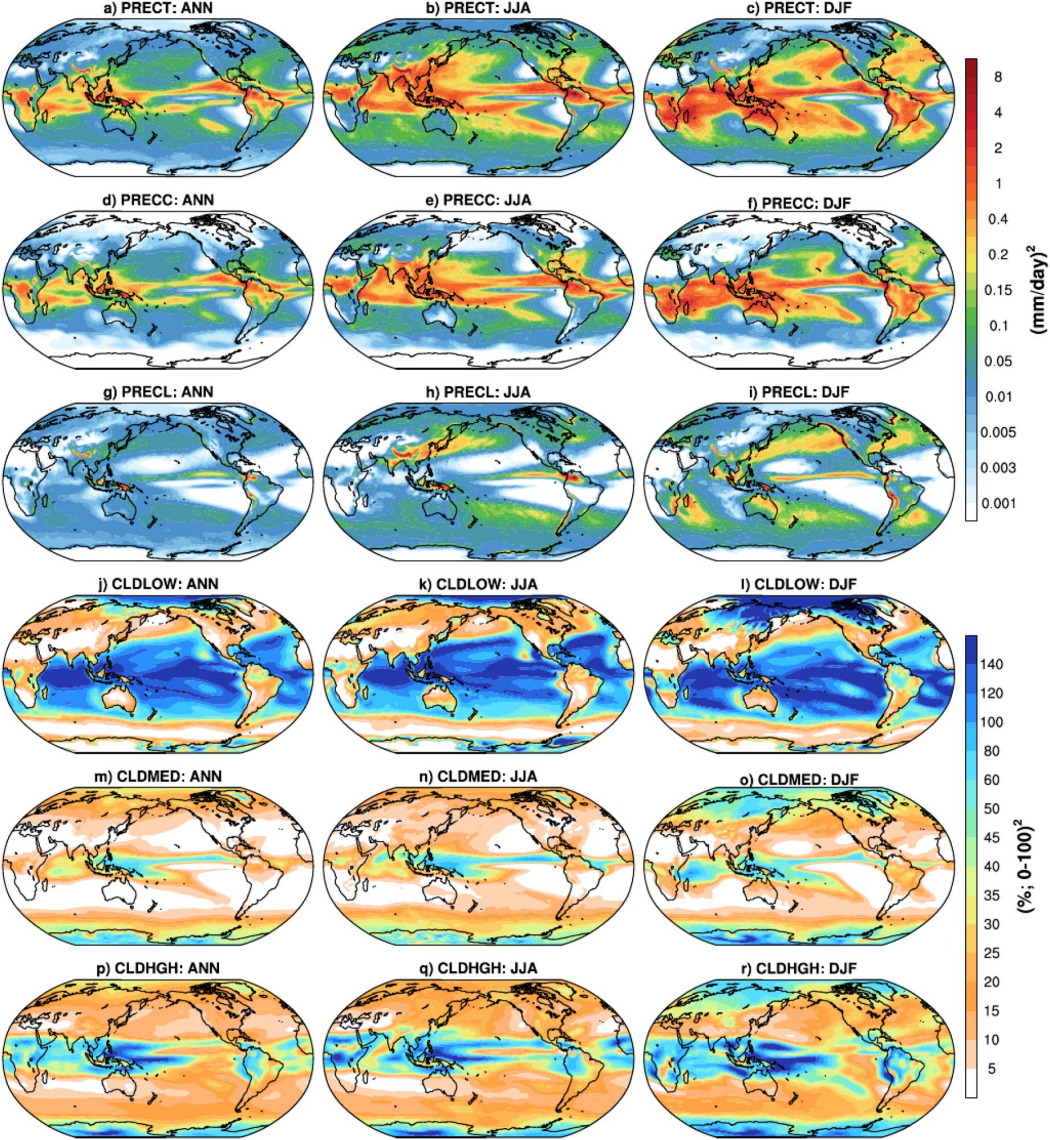
Global spatial inter-simulation variance distribution from 180 CAM5 model simulations from the perturbed parameter set for ANN, JJA, and DJF periods for mean PRECT, PRECC, PRECL, CLDLOW, CLDMED, and CLDHGH. NCAR Command Language (NCL) version-6.4.0 (scientific data analysis and visualization software; https://www.ncl.ucar.edu/) is used for computing the inter-simulation variance at each grid point and plotting over the global region.

Figure 1 shows the spatial variance distribution of annual (ANN), June–August (JJA), and December-February (DJF) mean precipitation (total, convective, and large-scale), and cloud fraction (low, medium, and high) from 180 model simulations. The variance in each of the different variables shown in Fig. 1 is a measure of the variability in the 180 simulations performed by perturbing the parameters within their upper and lower bounds. For the total precipitation (PRECT), large variance occurs over the tropics, South Pacific Convergence Zone (SPCZ), South America, Central Africa, and Himalaya during ANN (Fig. 1a). Qian et al.^23^ have also noticed large variance over the tropical regions during ANN from the perturbation of cloud and aerosol related parameters. On a seasonal scale, the variance in PRECT is relatively higher (~ 2 times) than that for ANN over the regions mentioned above, and over the Indian land, Indian ocean, eastern China, and subtropical northwestern Pacific region during JJA, and the subtropical northeastern Pacific and Atlantic stratus region during DJF (Fig. 1b,c). For the convective precipitation (PRECC), the region of large variance for ANN, DJF, and JJA is similar to PRECT, except over the subtropical Pacific and Atlantic region (in all periods) and the subtropical western Indian ocean (in DJF), where the large variance occurs in large-scale precipitation (PRECL) (Fig. 1d–i). Also, PRECC and PRECL are the primary contributors of PRECT over the tropical and subtropical regions, respectively^8,55^.

For the cloud fraction, large variance in low-level clouds (CLDLOW) occurs over the tropical oceanic and Arctic regions, and moderate to small variance occurs over the global continental land areas for ANN^32^, JJA, and DJF (Fig. 1j–l). It is to be noted that CLDLOW over the oceanic region is sensitive to the SST, but this study uses prescribed climatological SST and hence coupled model simulations could show somewhat different patterns. For medium-level clouds (CLDMED), large variance occurs over the equatorial region and small to moderate over the higher latitudes across the different periods (Fig. 1m–o). For high-level clouds (CLDHGH), the pattern of variance (Fig. 1p–r) over the tropical regions is similar to CLDMED; however, its magnitude over the tropical region is slightly higher than CLDMED and CLDLOW. In general, CLDHGH and CLDLOW contribute almost equally to the total cloud (CLDTOT), in line with the findings of Kay et al.^56^.

**Figure 2:**
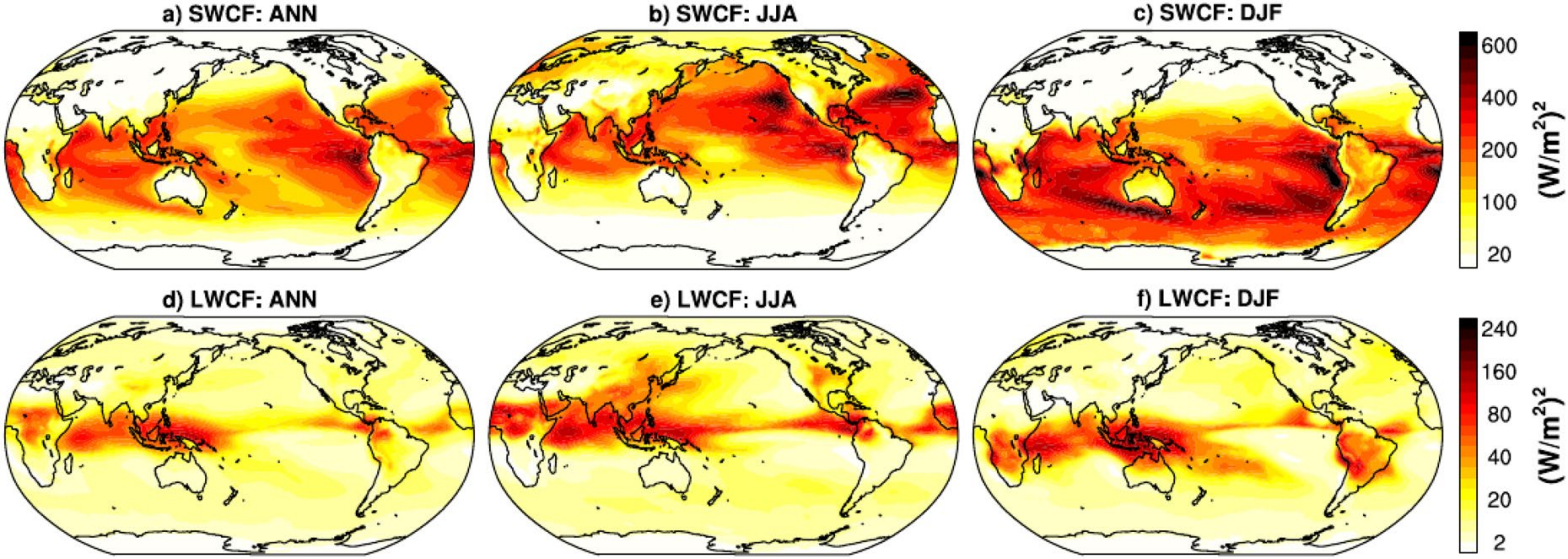
Global spatial inter-simulation variance distribution from 180 CAM5 model simulations from the perturbed parameter set for ANN, JJA, and DJF periods for mean SWCF and LWCF. NCAR Command Language (NCL) version-6.4.0 (https://www.ncl.ucar.edu/) is used for computing the inter-simulation variance at each grid point and plotting over the global region.

In addition, large variance in shortwave cloud forcing (SWCF) occurs over the tropical oceanic region and small to moderate over the continental land areas, with highest over the eastern Pacific, Atlantic, and Indian Ocean during ANN. The seasonal SWCF variance follows the sun seasonal migration (i.e., it is highest in JJA over the northern hemisphere’s oceanic region from 10° S to 40° N, and in DJF over the southern hemisphere’s oceanic region from 10° N to 40° S) (Fig. 2a–c). The smaller SWCF variance over land than the oceanic region is also reported from SA of cloud parameters in Lin et al.^57^ and Cloud Unified By Binormals (CLUBB) parameters in Guo et al.^32^ during ANN. The large SWCF over the subtropical region has been reported in previous studies due to persistent low-level stratus clouds^58^, and the SWCF bias over that region is reported to be a serious concern in existing CMIP5 models, and hence the unsatisfactory simulation of mid-latitude westerly jet^59^. Further, large variance in longwave cloud forcing (LWCF) occurs over the inter-tropical convergence zone (ITCZ), similar to the large PRECC variance region across different periods (Fig. 2d–f). Ardanuy et al.^58^ have reported that the large LWCF variability over the ITCZ region could be due to the high sensitivity of high-level tropical clouds associated with deep convection (Fig. 1).

### Sensitivity analysis for global climate simulations

#### Spatial distribution of relative importance

**Figure 3:**
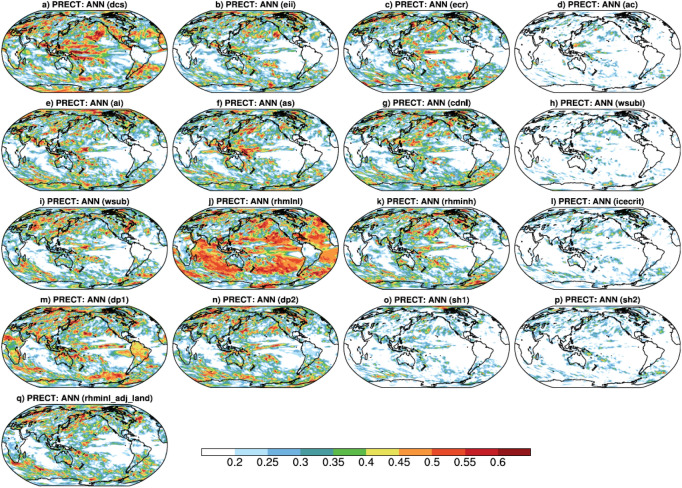
Spatial distributions of the RI of parameters for annual mean total precipitation (PRECT) simulation from 180 model simulations. PSUADE version-1.7.8b software (https://computing.llnl.gov/projects/psuade-uncertainty-quantification) is used for RI computation using the Morris method at each grid point, and NCAR Command Language (NCL) version-6.4.0 (https://www.ncl.ucar.edu/) is used for plotting over the global region.

Figure 3 shows the spatial distribution of relative importance (RI) of 17 uncertain parameters from the MOAT sensitivity analysis in the simulation of mean annual PRECT. We find highest RI of—(a) $rhminl$ over tropical oceanic and subtropical stratus region, (b) $dcs$ over SPCZ, North America, Europe, and northwestern Asia, (c) $as$ over Indo-Pacific warm pool and northwestern Pacific storm tracks region, and (d) $ai$ over the Arctic region, and northern parts of the Arabian Sea and Bay of Bengal (BoB). Some parameters show moderate RI, such as $dp1$ over the land areas of South America, Asia, Africa, and over the oceanic region of southern subtropical and equatorial Pacific, and $wsub$ over Central Asia. We also find parameters which show lesser spatial RI for ANN, but show seasonal dependence, such as, (a) $cdnl$ shows highest RI over the northeastern Pacific region, and moderate over South America and South Africa during JJA and northeastern Asia during DJF, (b) $dp2$ shows higher RI over northcentral Pacific during DJF, and (c) $ecr$ shows highest RI over the southern subtropical Pacific region during JJA (Supp. Figs. S1, S2).

Some of the most sensitive parameters in the annual mean spatial PRECT simulation reported here agree well with previous findings on SA. For example, Qian et al.^23^ have reported that the largest uncertainty in PRECT simulation over the oceanic region is primarily controlled by $rhminl$ and $dcs$. He and Posselt^39^ have shown $dcs$, $ai$, and $rhminl$ as the most sensitive parameters in the simulation of tropical cyclones. Covey et al.^38^ have also reported large sensitivity of $rhminl$ in global mean PRECT simulation. Plausible physical mechanisms through which the most sensitive parameters influence the annual and seasonal mean PRECT simulation and other important global climate variables are discussed in “Impact of parameters to global climate simulations” section.

#### RI of parameters on simulation of important climatic variables

**Figure 4:**
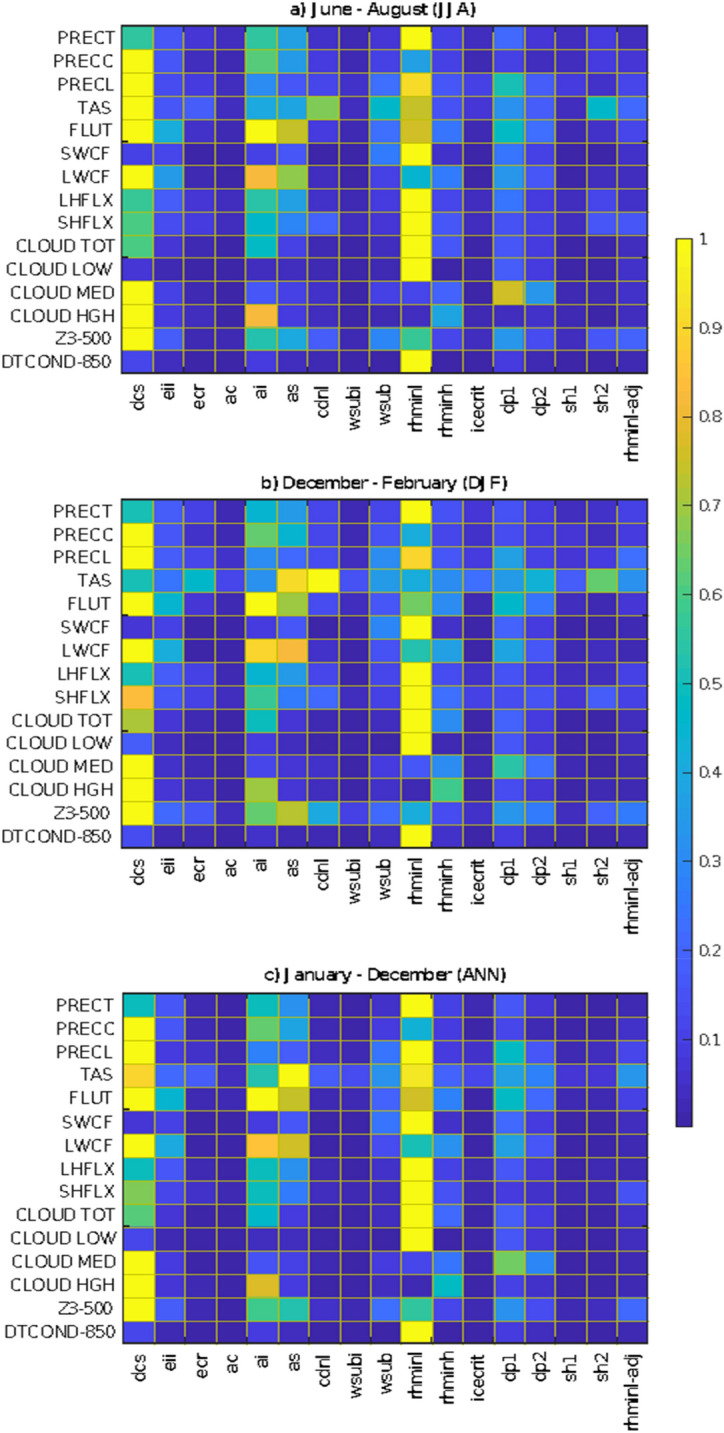
RI in the simulation of various climate variables over the global region during (**a**) JJA, (**b**) DJF, and (**c**) ANN, using the Morris method. The y-axis shows the various climate variables, and the x-axis shows various parameters used in the sensitivity study. A higher value is indicative of higher sensitivity (see Table 3 for details on parameters and Table 1 for details on variables).

In this section, we first discuss the RI of specific parameters on simulation of important climatic variables at the global scale, monsoon regions, and storm track regions during ANN, JJA and DJF (see Table 1 list of variables), and then the plausible mechanisms through which these variables get affected. In this regard, from Fig. 4, we find the global mean PRECT simulation is most sensitive to parameters that reported highly sensitive in spatial PRECT pattern^23,37^ (Fig. 3). However, PRECC and PRECL simulation are highly sensitive to ($$rhminl, dcs, ai, as, dp1)$$, and ($$dcs, rhminl, ai, as, dp1)$$, respectively. Please note in the text the sensitivity of parameters is discussed in decreasing order of sensitivity. Further, CLDLOW, CLDMED, CLDHGH, and CLDTOT simulation is most sensitive to $$\left( {rhminl} \right)$$^37^, ($$dp1,dcs)$$, ($$ai, dcs)$$ and $$\left( {rhminl, dcs, ai} \right)$$, respectively, across the different periods. The large sensitivity of $$rhminl$$ in CLDLOW simulation was also reported in Zhang et al.^20^. The SWCF, LWCF, latent heat flux (LHFLX), and sensible heat flux (SHFLX) simulation is most sensitive to $$\left( {rhminl} \right)$$, ($$dcs, ai, as)$$, $$\left( {rhminl} \right)$$ and $$\left( {rhminl} \right)$$ during all periods, respectively. The outgoing longwave flux (FLUT) is highly sensitive to $$\left( {dcs, ai, as} \right)$$ during JJA and ANN, while it becomes slightly less sensitive to ($$as)$$ during DJF. Furthermore, from Fig. 4, we notice that the simulation of surface air temperature (TAS) is most sensitive to ($$dcs$$, $$rhminl$$, $$cdnl$$, $$sh2$$) during JJA, to ($$cdnl$$, $$as$$, $$sh2)$$ during DJF, and to ($$rhminl$$, $$dcs, ai)$$ during ANN.

**Table 1:** List of climate variables used to assess simulation sensitivity.

Sr. no.	Variable	Description	Unit
1.	PRECT	Total (convective + large-scale) precipitation rate	mm/day
2.	PRECC	Convective (deep + shallow) precipitation rate	mm/day
3.	PRECL	Large-scale precipitation rate	mm/day
4.	TAS	Surface air temperature	K
5.	FLUT	Upwelling longwave flux at the top of the model	W/m^2^
6.	SWCF	Shortwave cloud forcing	W/m^2^
7.	LWCF	Longwave cloud forcing	W/m^2^
8.	LHFLX	Surface latent heat flux	W/m^2^
9.	SHFLX	Surface sensible heat flux	W/m^2^
10.	CLDTOT	Vertically-integrated total cloud	Fraction (0–1)
11.	CLDLOW	Vertically-integrated low cloud (lower than 700 hPa)	Fraction (0–1)
12.	CLDMED	Vertically-integrated mid-level cloud (700 to 400 hPa)	Fraction (0–1)
13.	CLDHGH	Vertically-integrated high cloud (400 to 50 hPa)	Fraction (0–1)
14.	TMQ	Total precipitable water	kg/m^2^
15.	Z3-500	Geopotential height at 500 hPa	meter
16.	DTCOND-850	T tendency—moist processes at 850 hPa	K/s
17.	LWP	Liquid water path	kg/m^2^
18.	IWP	Ice water path	kg/m^2^

**Figure 5:**
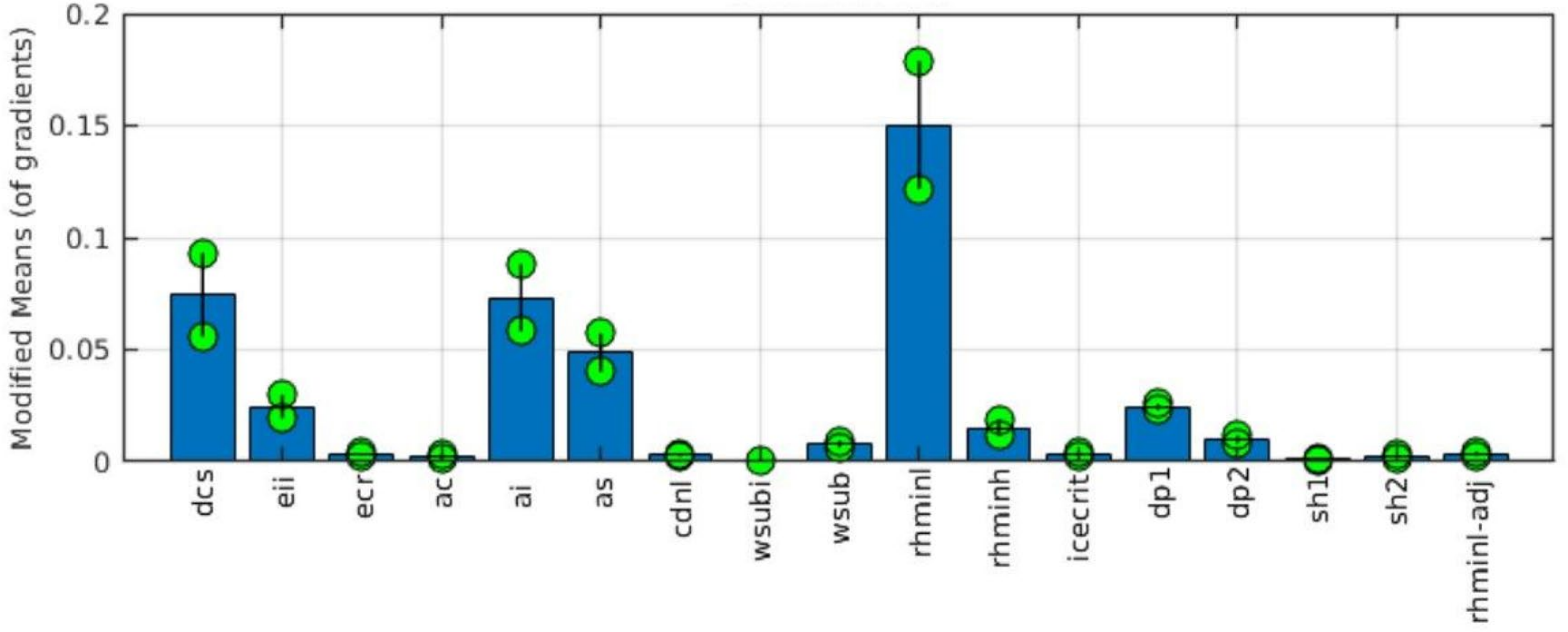
The bars show first-order sensitivity (main effect) in the simulation of total precipitation over the global region during ANN using the Morris method. The marker line spread on the bar graph shows sensitivity due to interactive effects with other parameters. Large values of modified means of gradients and marker line spread of a parameter are indicative of higher sensitivity.

The RI of specific parameters on global climate simulation seen in Fig. 4 can be better understood in Fig. 5. In general, parameters with a large value of modified means (µ) are considered to have large main effects, and parameters with a large value of standard deviation (σ) are considered to have large interactive effects (i.e., interactions with other parameters^38^). However, parameters with a small value of µ and σ are considered less sensitive, and parameters with a large value of µ and σ are considered most sensitive (which is usually referred to as RI^60^). Thus, we find that for PRECT simulation, ($$rhminl$$, $$dcs$$, $$ ai$$, $$as$$) are most sensitive, with very large main and interactive effects during all periods. Morris results also show few parameters, such as collection efficiency of ice aggregation ($$eii$$), deep convection cloud fraction ($$dp1$$), and the threshold relative humidity parameter for high clouds ($$rhminh)$$ with small to moderate level of sensitivity to PRECT simulation, and rest are highly insensitive. The spatial patterns of main and interactive effects of all parameters in PRECT simulation are also shown in Supp. Figs. S3 and S4.

**Figure 6:**
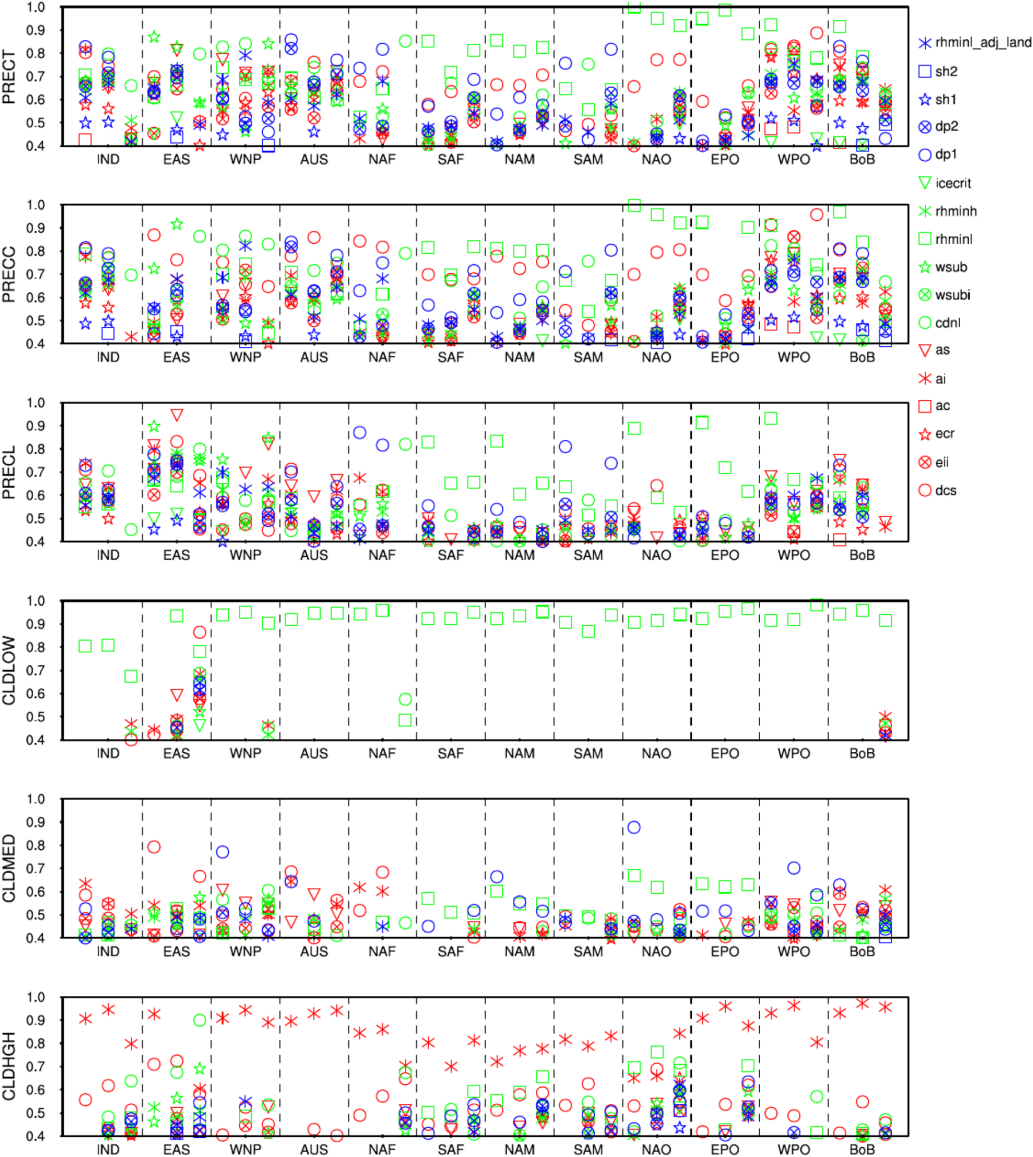
The RI measure in the simulation of PRECT, PRECC, PRECL, CLDLOW, CLDMED, and CLDHGH over the global monsoons and storm track regions (see Table 2 for latitude/longitude). Each box in a panel shows three different periods—ANN, JJA, and DJF. The y-axis shows the RI, and the x-axis shows various domains for ANN, JJA, and DJF. A higher value of RI is indicative of higher sensitivity.

We also report the seasonal and regime-specific dependence of some parameters for the monsoon and storm track regions (see Table 2 for latitude and longitude details), such as local summer precipitation over the monsoon region of India, Australia, North Africa, South America and the storm track region of BoB are most sensitive to $dp1$. In contrast, the local winter precipitation over—India is more sensitive to $wsub$, Australia is more sensitive to $dcs$, North Africa and South America are more sensitive to $cdnl$ (Fig. 6). The western Pacific storm track region also shows seasonal dependence; it is more sensitive to $cdnl$ in JJA, whereas more sensitive to $wsub$ in DJF. The large sensitivity of $dp1$ over the monsoon region is evident due to the large convective activity during the summer period^52,61,62^. However, irrespective of the regions and seasons, CLDLOW and CLDHGH are most sensitive to $rhminl$ and $ai$, respectively (Fig. 6).

**Table 2:** Details of the various monsoons and storm track regions (see Bal et al.^63^ for more information).

Sr. no.	Monsoon regions	Longitude; latitude
1.	Indian monsoon (IND)	67–97 E; 10–35 N
2.	East Asian monsoon (EAS)	115–125 E; 28–46 N
3.	Australian monsoon (AUS)	110–150 E; 5–15 S
4.	Western north Pacific monsoon (WNP)	110–115 E; 10–28 N
5.	North African monsoon (NAF)	25–30 W; 5–15 N
6.	South African monsoon (SAF)	5–90 E; 0–30 S
7.	North American monsoon (NAM)	50–125 W; 0–30 N
8.	South American monsoon (SAM)	30–80 W; 5–30 S

	**Storm track regions**	
9.	North Atlantic Ocean (NAO)	45–75 W; 10–30 N
10.	East Pacific Ocean (EPO)	85–150 W; 10–20 N
11.	West Pacific Ocean (WPO)	120–150 E; 10–30 N
12.	Bay of Bengal (BoB)	75–105 E; 5–25 N

#### Impact of parameters to global climate simulations

**Figure 7:**
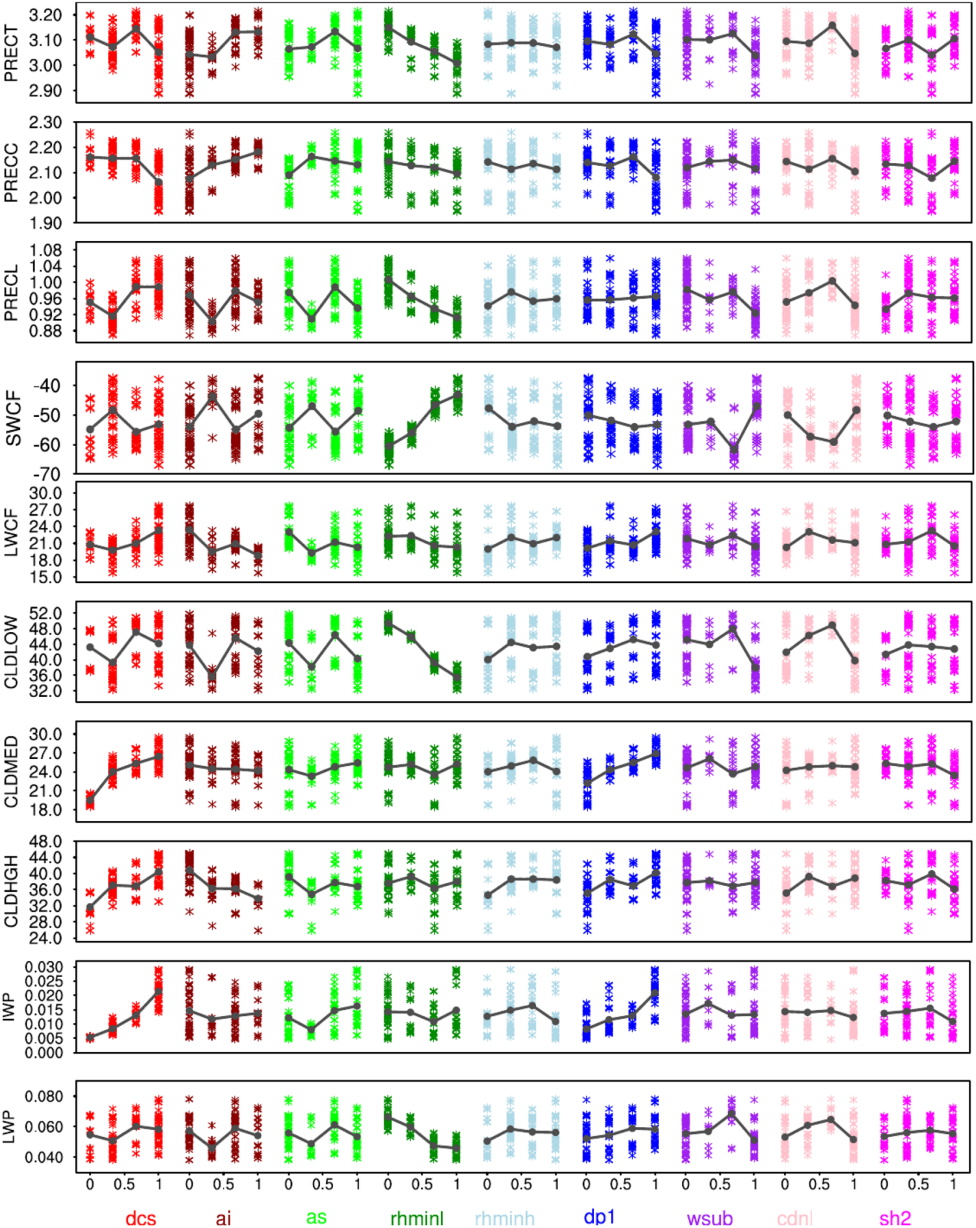
The response of various important global annual mean climate variables (PRECT, PRECC, PRECL, SWCF, LWCF, CLDLOW, CLDMED, CLDHGH, IWP, and LWP) to perturbations in some of the most sensitive cloud parameters from 180 model simulations. A solid dark gray line shows the average effect for a parameter. See Table 1 for the full name of the variables and unit details.

**Figure 8:**
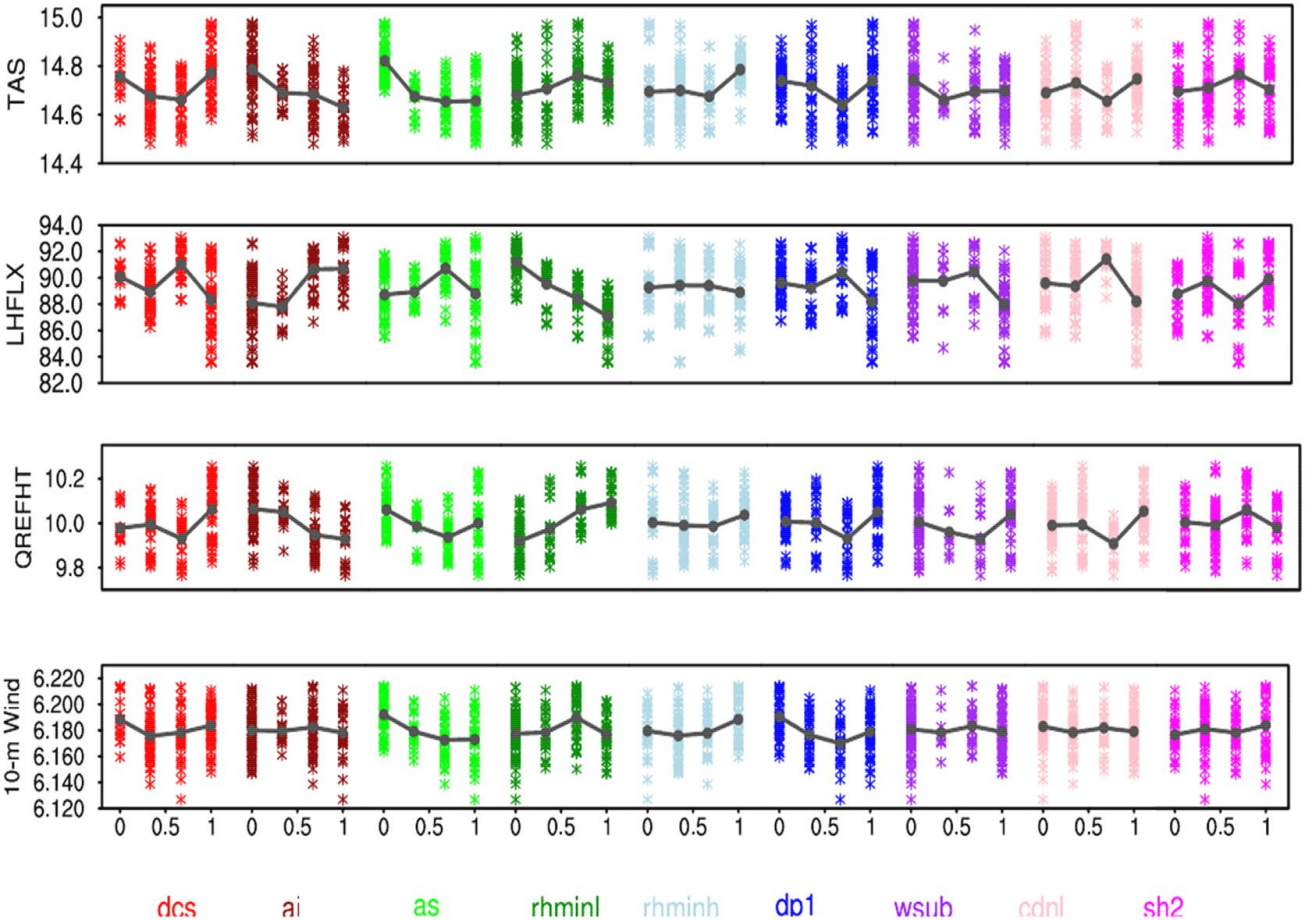
The response of global annual mean TAS, LHFLX, 2-m specific humidity (QREFHT), and 10-m wind (10-m Wind) to the perturbation of parameters from 180 model simulations. A solid dark gray line shows the average effect for a parameter.

Furthermore, in response to the perturbation of various important parameters, we present the global annual mean variation of different climatic variables in Figs. 7 and 8. We find PRECT to decrease monotonically from ~ 3.15 to 2.95 mm/day in response to increase in $$rhminl$$, and increase monotonically from ~ 3.0 to 3.15 mm/day in response to an increase in $$ ai$$, which is in line with the findings of Qian et al.^23^ (Fig. 7). Since $$rhminl$$ directly affects the amount of stratiform low cloud, increased $$rhminl$$ decreases the CLDLOW (from ~ 50 to ~ 35%), which then lowers the SWCF (from ~  − 65 to − 45 W/m^2^) (Fig. 8). The linear decrease in LHFLX (from ~ 92 to 86 W/m^2^) in response to an increase in $$rhminl$$ can be seen as a result of an increase in near-surface (2-m) specific humidity. The increase in near-surface specific humidity with an increase in $$rhminl$$ could be linked to the fact that increasing the $$rhminl$$ makes it difficult for moisture to precipitate out in the form of stratiform precipitation (PRECL), and hence leading to a buildup of low-level moisture. The decrease in PRECL with an increase in $$rhminl$$ can be seen in Fig. 7. The increase in near-surface specific humidity along with an increase in relative humidity (since TAS does not change much with $$rhminl$$) is causing a reduction in the surface evaporation (could be seen from the decrease in LHFLX) (Fig. 8). The linear decrease in liquid water path (LWP) with an increase in $$rhminl$$ is also found in some previous studies^23,33,34^ (Fig. 7). In line with the findings of Lin et al.^57^, the decrease in PRECC to an increase in $$rhminl$$ is also seen by indirectly affecting the stability of the low-level atmosphere and hence the convection (Fig. 7).

We find opposite responses of $dcs$ and $ai$, and both show a large response to CLDHGH. In general, the increase in $dcs$ leads to lesser conversion of ice to snow, so more ice clouds remain in the upper troposphere^39^, and hence more ice water path (IWP) and LWCF. Since the increase in ice clouds makes the atmosphere more stable, there is a reduction in convective activity, and hence, a decrease in PRECC and PRECT. The increase in $ai$, however, leads more ice particles to fall, and therefore a decrease in CLDHGH, which then lowers LWCF and IWP^64^. As a result of the reduction in LWCF, TAS decreases mildly due to reduced greenhouse effect^33,49,58^. Falling of more ice with increasing $ai$ causes more precipitation (PRECT and PRECC), most likely due to increased convective instability^65,66^. The effect of $dcs$ and $ai$ on SWCF is small and non-linear, in line to the response of $as$, which acts quite similar to $ai$, but for snow.

In addition, we also find non-monotonic behavior of $cdnl$, which, in general, increases cloud droplet number concentration with increasing $cdnl$ through the increased activation of smaller aerosol particles^27,67^. The strong response of $cdnl$ is found in the simulation of CLDLOW and hence, SWCF, such that the global mean annual SWCF increases with a decrease in $cdnl$ and vice-versa. An increase in CLDLOW is found consistent with an increase in LWP and PRECL. The response of CLDHGH to $cdnl$ is minimal, and the same is the case for LWCF. More specifically, $cdnl$ affects SWCF by increasing the albedo from the increased number of cloud concentration and liquid clouds lifetime. Sensitivity to $wsub$ perturbations is similar to $cdnl$, but smaller in amplitude. Since $cdnl$ and $wsub$ strongly influence the liquid clouds^35^, they have a relatively lower impact on TAS than other parameters. We also find the linear response of $dp1$ to CLDHGH, increasing $dp1$ increases CLDHGH, which then causes the increase in IWP and LWP, and hence LWCF. The increase in $dp1$ also increases CLDLOW substantially, and hence SWCF.

### Sensitivity analysis for SASM features

#### RI of parameters on simulation of SASM features

Model biases over South Asia during the summer monsoon are very large, not only in this model but also in many of the CMIP5 models^68,69^. The spatial precipitation distribution, onset and withdrawal, and interannual and intraseasonal variability are some important SASM features and are not satisfactorily simulated in existing models^51,68,69^. Previous findings suggest that the skill of SASM simulation is largely dependent on a model’s ability to simulate the strength of the low-level Somali jet (SJS), upper-level tropical easterly jet (TEJ), inter-tropical convergence zone (ITCZ), local Hadley circulation strength (HCS), meridional tropospheric temperature gradient (MTTG), and the easterly vertical shear of zonal wind (ESZW) over the Indian region^6,51,52,68^. Hence, we further attempt to understand how the sensitive parameters in cloud parameterizations affect the SASM simulation. In this respect, we first find the most sensitive parameters related to some essential features of SASM using MOAT SA and then present the likely physical mechanisms causing such sensitivity.

**Figure 9:**
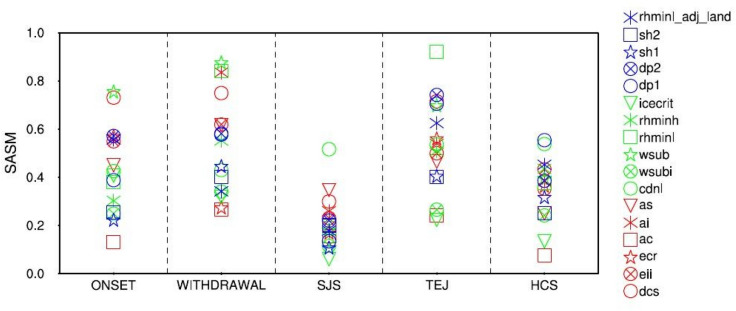
RI in the simulation of the various important SASM features during JJAS, such as onset, withdrawal, SJS, TEJ, and HCS. The y-axis shows the RI, and the x-axis shows various important SASM features. A higher value of RI is indicative of higher sensitivity.

Figure 9 shows the RI of sensitive parameters on the simulation of monsoon onset, withdrawal, SJS, TEJ, and HCS. SASM onset over India is defined when MTTG changes from negative to positive value on an annual cycle, and vice-versa for the withdrawal date^52,70,71^. MTTG is calculated by subtracting the vertically (600–200 hPa) averaged air temperature over BOX2 (5° N–15° S; 40°–100° E) from that of BOX1 (5°–35° N; 40°–100° E). We find the SASM onset over India to be highly sensitive to ($$wsub, dcs, dp1, as, rhminl$$), and withdrawal to be highly sensitive to $$\left( {wsub, rhminl,ai, dcs, as, dp1} \right)$$. The SJS, defined as the weighed-area-averaged speed over (15° S–15° N; 37.5°–62.5° E)^72^, is highly sensitive to ($$cdnl$$, $$as, ai, dcs, rhminl, dp1$$). Also, the TEJ, defined as the wind speed at 150 hPa along 10° N from 40°–110° E, is highly sensitive to ($$rhminl, dp1, wsub,dcs)$$. Further, we find the HCS, defined as the difference in the weighted-area-averaged meridional wind strength between 850 and 200 hPa over (70°–105° E; 5°–30° N)^73^ is mildly sensitive to ($$dp1,cdnl, eii$$).

#### Impact of parameters to SASM features

**Figure 10:**
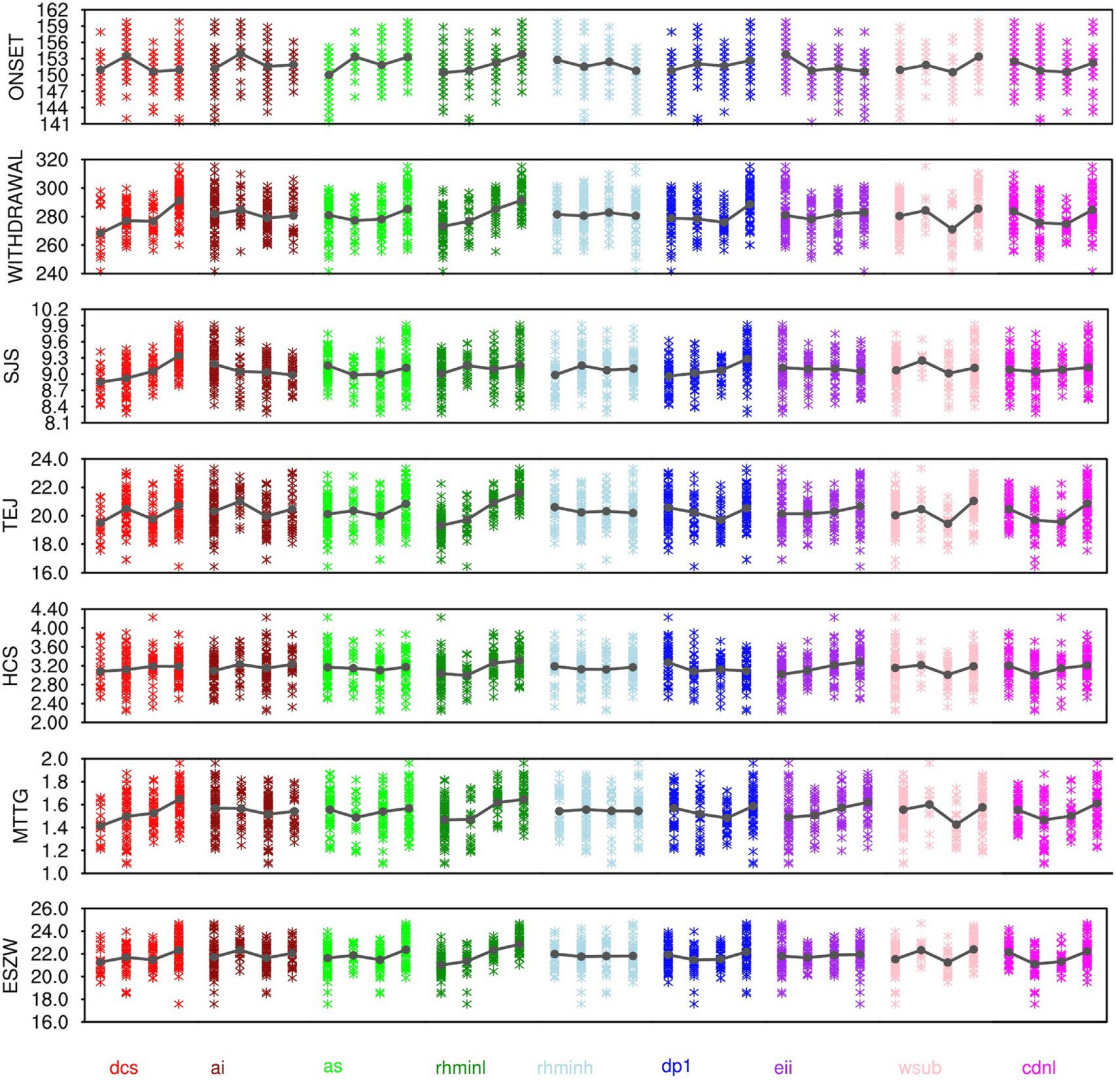
The response of various important SASM features to the perturbation of parameters from 180 model simulations. The important features are—ONSET, WITHDRAWAL, SJS, TEJ, HCS, MTTG, and ESZW. A solid dark gray line shows the average effect of a parameter.

Figure 10 shows the perturbation response for different parameters in the simulation of onset and withdrawal dates, SJS, TEJ, HCS, and some other dynamical features responsible for maintaining SASM. We find that the onset date increases monotonically with $rhminl$ (up to ~ 5 days), $dp1$ (up to ~ 4 days), $as$ (up to ~ 5 days), decreases with $eii$ (up to ~ 4 days), and responds non-monotonically to $dcs$, $ai$, and $cdnl$. The withdrawal date increases monotonically with $dcs$ (up to ~ 22 days), $rhminl$ (up to ~ 15 days), and responds non-monotonically to $wsub$ and $cdnl$.

The onset and withdrawal dates, strength, and sustenance of SASM are noted in previous studies to be strongly influenced by MTTG^68,70,73,74^. In this regard, we find that the MTT (BOX1) is increasing substantially with $$dcs$$ (up to ~ 1 °C), and $$rhminl$$ (up to ~ 0.5 °C), and decreasing with $$ai$$ (up to ~ 1 °C). It shows a non-monotonic response to $$as $$ and $$ dp1$$. We also find that the response of MTT (BOX2) is similar to MTT (BOX1) (Supp. Fig. S5). Hazra et al.^51^ have reported that the MTTG simulation reliability from GCMs largely depends on the reliability of the simulation of vertical cloud distribution. In line with the results in “Impact of parameters to global climate simulations” section for global annual means, we find that LWCF increases with increase in $$dcs$$ and $$dp1$$, and decreases with increase in $$ai$$ and $$rhminl$$ over BOX1 and BOX2 (with different amplitudes of response; Supp. Figs. S5, S6). SWCF also shows a similar response to parameters as noted in LWCF (except for $$rhminl$$ over BOX1, and $$dcs$$ and $$ai$$ over BOX2, that do not show any significant changes). The uneven changes in LWCF and SWCF over BOX1 and BOX2 to parameter perturbations modulate the vertical temperature distribution unevenly over BOX1 and BOX2 and hence change the MTTG simulation, which then affects the onset and withdrawal, and the strength of SASM (Fig. 10).

We further find that SJS monotonically increases with $dcs$ and $dp1$, and decreases with $ai$, and non-monotonically responds to $as$. TEJ monotonically increases with $rhminl$, and non-monotonically responds to $dcs$, $cdnl$, $dp1$, and $wsub$. HCS shows only a mild increase with an increase in $rhminl$ and $eii$, and a mild decrease with an increase in $dp1$, and responds non-monotonically to $cdnl$. In addition, the ESZW calculated as a difference of wind strength between 850 hPa (EW_850) and 200 hPa (EW_200) over (0°–15° N; 50°–90° E) also influences SJS (Fig. 10). The ESZW strength exceeding 20 m/s during JJAS was reported to be critical for maintaining the SASM^52,53^. ESZW is affected by the parameter perturbations through the changes in the vertical temperature and cloud distribution. We find that the ESZW monotonically increases with an increase in $rhminl$ (up to ~ 3 m/s) and non-monotonically responds to $dp1$ and $cdnl$. Specifically, we find that EW_850 increases substantially with $dcs$ (up to ~ 2 m/s), $rhminl$ (up to ~ 1.5 m/s), and $dp1$ (up to ~ 1 m/s). However, EW_200hPa decreases substantially with an increase in $rhminl$, and non-monotonically responds to $dp1$ and $cdnl$ (Supp. Fig. S5).

## Conclusions

In this study, we have performed the sensitivity analysis of 17 parameters from the cloud microphysics and macrophysics parameterizations used in the NCAR CAM5. Morris method has been used to identify parameters that have large main and interactive effects on the global climate simulation, global monsoons (with a special emphasis on SASM), and storm track regions on an annual and seasonal basis.

We find the highest variability in PRECT over the tropical convective region and moderate variability in PRECT over the subtropical stratiform region, which arise due to the large variance in PRECC and PRECL, respectively, from 180 inter-parameter perturbed simulations^8,55^. Also, the large variance in LWCF, CLDHGH, CLDMED, total precipitable water (TMQ), LWP, and IWP are found over the large PRECT variance region^39,58^. The large variance in LWP and IWP over the tropical and subtropical regions could be one of the reasons for the large variance in PRECT simulation^32^. For the annual mean spatial PRECT simulation, we find the highest spatial RI of the following parameters: $rhminl, dcs, as, ai, dp1, wsub, and cdnl,$ across the different periods. RI finding reported here is in line with previous studies for some parameters, such as $rhminl$ and $dcs$ are reported most sensitive over the tropical regions^23^, $dcs$, $ai$, and $rhminl$ are reported most sensitive over the western Pacific^39^, and $rhminl$ is reported most sensitive over the global region on PRECT simulation^38^. In addition, we find large main and interactive effects of $rhminl$, and moderate main and interactive effects of $dcs$, $ai$, and $as$ on PRECT simulation^23,37^. Parameters $rhminl$ and $dcs$ are most sensitive for PRECC and PRECL simulations, respectively.

Simulations of cloud fraction (total, low, mid, and high) are very sensitive to $rhminl$ and $dcs$. Zhang et al.^20^ have also reported the large $rhminl$ sensitivity and suggested that $rhminl$ tuning can better simulate the clouds in CAM. Simulation of SWCF and LWCF are most sensitive to $rhminl$, and $dcs$ and $ai$, respectively. Simulation of SHFLX and LHFLX are most sensitive to $rhminl$ and $dcs$. Notably, we find seasonal dependence in the sensitivity of some parameters, for example, in the simulation of TAS, $dcs$ and $rhminl$ are most sensitive during JJA, whereas $as$ and $cdnl$ are most sensitive during DJF. In addition to the seasonal dependence, we find regime specific dependence of some parameters, such as the summer precipitation over the different monsoon and storm track regions are more sensitive to $dp1$, while the winter precipitation does not show any systematic sensitivity. The large sensitivity of $dp1$ during the summer period over the monsoon region could be due to the presence of large convective clouds^52,61,62,65^.

In response to parameter perturbation, we find a monotonic decrease in PRECT with $rhminl$ due to reduced low-level stratiform and shallow cumulus clouds and hence the reduced LWP and SWCF^20,32,33^. We find an increase in LWCF and IWP with $dcs$ as the increase in $dcs$ leaves more ice clouds at high-level, thereby decreasing the stability of the atmosphere and hence the decrease in PRECC and PRECT. We also find that the response of precipitation simulation to $ai$ and $as$ is opposite to $dcs$—increase in $ai$ ($as$) causes more ice (snow) particles to fall; thus, it reduces CLDHGH, LWCF, and IWP^64–66^. With the increase in $cdnl$ and $wsub$, there is an increase in PRECL and LWP due to an increase in stratiform low clouds and low-level cloud droplet concentration, respectively.

Further, analyzing the SASM simulations, we find that the monsoon onset and withdrawal dates^68,69^ are highly sensitive to some of the cloud parameters through their considerable influence on MTTG. In general, we find that the sensitivity of MTTG occurs via changes in vertical cloud distribution and cloud radiative forcings^51^. We find MTTG to be most sensitive to $dcs$, due to the higher sensitivity of MTT (BOX1) to $dcs$, as there are more high-level clouds during SASM over BOX1 (Supp. Figs. S5, S6). We find SJS to be most sensitive to $dcs$. According to Krishnamurti et al.^74^, SJS is strongly related to the tropospheric temperature over the Indian land (roughly represented by MTT (BOX1)). The increase in $dcs$, increases MTT (BOX1) by increasing the cloud forcings (Supp. Figs. S5, S6), and hence increases SJS. We also find that ESZW and TEJ are most sensitive to $rhminl$, and caused by the higher sensitivity of EW_200hPa than EW_850hPa to $rhminl$ (Supp. Fig. S5). The higher sensitivity of EW_200hPa could be due to high sensitivity in the strength of the Tibetan anticyclone, thus leading to higher sensitivity in the sub-tropical westerly jet. The high sensitivity of EW_200hPa also explains the high sensitivity of the TEJ to $dcs$. We do not find HCS to show high sensitivity to any of the parameters discussed above.

Thus, through the identification of the most sensitive parameters in the cloud parameterization schemes used in the NCAR CAM5, our study helps prioritize tuning efforts, not only for the model used in this work but also for any global or regional model using similar parameters in their cloud parameterization schemes. Informed tuning efforts complemented by observational constraints to the most sensitive parameters used in climate models will be crucial for reducing parameter-sensitivity induced uncertainty in both historical climate simulations and climate change projections.

## Methodology

### Model description and simulation details

NCAR CAM5 under the framework of CESM1.2.2 has been used to carry out the model simulations for sensitivity analysis. CAM5 uses the finite volume dynamical core from Lin and Rood^75^ and Lin^76^, shallow convection scheme from Park and Bretherton^77^, deep convection scheme from Zhang-McFarlane^78^ with modifications to include dilute parcel computations by Neale et al.^79^, momentum transport by Richter and Rasch^80^. Also, CAM5 uses the radiation scheme from Iacono et al.^81^ and Mlawer et al.^82^, two-moment cloud microphysics scheme from Morrison and Gettelman^83^, and Gettelman et al.^84^, cloud macrophysics scheme from Park et al.^85^, and moist turbulence scheme from Bretherton and Park^86^. For more details on CAM5 configuration, see Neale et al.^87^.

We have conducted a set of 180 simulations for 6-year each (a total of 1080 years of model integration) at a horizontal resolution of 0.9° latitude × 1.25° longitude and 30 vertical levels, using prescribed monthly climatological sea surface temperature (SST). The last five years of the simulations are used in this analysis, discarding the first year as spin-up. The average values of the selected climate variables (see Table 1) for each 5-year simulation on annual (ANN) and seasonal (i.e., for June–August (JJA) and December–February (DJF)) time-scales are considered as one sample for ANN, JJA, and DJF, respectively.

### Parameters

Parameters from cloud microphysics and macrophysics parameterization schemes are used in this study (see Table 3), and the climate variables used to assess sensitivity are shown in Table 1. Generally, there are many uncertain parameters in cloud physics parameterizations; we choose to highlight the sensitivity analysis for those parameters that are not explored much in the regional and seasonal context. We used 17 parameters: 9 from cloud microphysics and 8 from cloud macrophysics schemes (see Table 3 for lower and upper bound). The 15 parameters (out of 17 parameters) have been chosen from a large set of parameters explored in the existing literature^23,33,38,39^. The default values, lower, and upper limits of these parameters are taken from previous studies^23,39^. For the two additional parameters related to shallow cloud fraction, the lower and upper bounds are taken from the 95% interval of high posterior probability from our SCAM5 results (and this range matches to ~ 50% on both sides of the default value).

**Table 3:** List of cloud microphysics and macrophysics parameters used in this study.

Parameter name	Description	Range			Remarks
		Low	Default	High	
**Parameters in cloud microphysics**					
dcs	Auto-conversion size threshold for ice to snow	1e−4	2.5e−4	5e−4	Affect mainly to the high cloud distribution; higher $dcs$-value corresponds to the lesser conversion of cloud ice to snow
eii	Collection efficiency aggregation ice	0.001	0.1	1.0	Affect mainly to the ice water content
ecr	Collection efficiency, accretion of cloud water by rain	0.5	1.0	1.5	Affect mainly to the cloud liquid water content
ac	Fall speed parameter for cloud water	1.5e+7	3e+7	4.5e+7	Affect mainly to the cloud water content
ai	Fall speed parameter for stratiform cloud ice	350	700	1400	Affect mainly to the ice water content
as	Fall speed parameter for stratiform snow	5.860	11.72	23.44	Affect the snow and the ice water content; larger $as$-values corresponds to the larger cloud water-fall speed
cdnl	cloud droplet number limiter	0	0	1e−6	Affect mainly to the cloud droplet number concentration
wsubi	Minimum subgrid vertical velocity for ice nuclei	1e-6	0.001	0.2	Affect mainly to the cloud droplet number concentration
wsub	Minimum subgrid vertical velocity for liquid nuclei	0	0.2	1.0	Affect mainly to the cloud droplet number concentration
**Parameters in cloud macrophysics**					
rhminl	Threshold relative humidity for stratiform low clouds	0.80	0.8875	0.99	Affect mainly to the low clouds; higher $rhminl$-value corresponds to the lesser low-level stable clouds below 700 hPa
rhminh	Threshold relative humidity for stratiform high clouds	0.65	0.80	0.85	Affect mainly to the high clouds; higher $rhminh$-value corresponds to the lesser high-level stable liquid clouds above 400 hPa
rhminl-adj-land	rhminl adjustment for snow-free land	0.05	0.10	0.15	Affect mainly to the low-level clouds over the snow-free land
icecrit	Critical relative humidity for ice clouds	0.47	0.93	1.4	Affect mainly to the ice clouds; higher $icecrit$-value corresponds to lesser ice clouds
dp1	Parameter for deep convection cloud fraction	0.05	0.10	0.15	Affect mainly to the deep convective cloud fraction
dp2	Parameter for deep convection cloud fraction	250	500	750	Affect mainly to the deep convective cloud fraction
sh1	Parameter for shallow convection cloud fraction	0.02	0.04	0.06	Affect mainly to the shallow convective cloud fraction
sh2	Parameter for shallow convection cloud fraction	250	500	750	Affect mainly to the shallow convective cloud fraction

### Morris method based sensitivity analysis

The Morris method based on MOAT sampling^17^ is used to quantify the elementary (main) effects of the parameters. This method can identify parameters whose effects may be negligible or linear or non-linear (or involved in interactions with other parameters). For each parameter, two global sensitivity measures (µ and σ) are computed, where µ shows the overall influence of the parameter on model output (climate variables) and σ shows higher-order effects (i.e., interactions with other parameters) of the parameter on climate variables. In the Morris method, each input parameter is varied across ‘$m$’ levels in the parameter space. The input parameters are selected from set $\left\{ {0, 1/\left( {m - 1} \right), 2/\left( {m - 1} \right), \ldots , 1} \right\}$ (in our case $m = 4$), and a single parameter is perturbed by $\Delta = \left( {m/2} \right)/\left( {m - 1} \right)$. To compute the elementary effect, let us consider $k$ parameters (in our case $k = 17$), and a random sample $S_{1} = \left\{ {x_{1} ,x_{2} , \ldots ,x_{k} } \right\}$ and another sample $S_{2} = \left\{ {x_{1} ,x_{2} , \ldots , x_{i} + \Delta_{i} , \ldots ,x_{k} } \right\}$ by perturbing the *i*th parameter by $\Delta_{i}$. The elementary effect of *i*th parameter $x_{i }$ is defined as $E_{i} = \frac{{\left\{ {f\left( {S_{2} } \right) - f\left( {S_{1} } \right)} \right\}}}{{\Delta_{i} }}$, where $f$ stands for the weighted-area-averaged value of a particular climate variable for which sensitivity effect will be analyzed. However, the $E_{i}$ computed here has the unit of $f/\Delta$ (which varies based on the parameter unit and variable unit) and can not be directly used for the comparison and ranking between the variables and other parameters. It is thus normalized according to Covey et al.^38^ as $E_{i} = \frac{{\left\{ {f\left( {S_{2} } \right) - f\left( {S_{1} } \right)} \right\}/f\left( {S1} \right)}}{{\Delta /\left( {x_{h} - x_{l} } \right)_{i} }}$, where $x_{h}$ and $x_{l}$ refer to the high and low values of a particular parameter range. Further, a third sample $S_{3} = \left\{ {x_{1} ,x_{2} , \ldots ,x_{i} + \Delta_{i} , \ldots ,x_{j} + \Delta_{j} , \ldots ,x_{k} } \right\}$ is generated by perturbing another parameter from remaining $k - 1$ parameters $\left( {j\, {\text{is not equal to}}\, i} \right)$, and by repeating this process until all the parameters are varied, we get $k + 1$ samples $\left( {S_{1} ,S_{2} , \ldots ,S_{k + 1} } \right),$ collectively called a single trajectory, and $k$ elementary effects ($E_{1} ,E_{2} , \ldots ,E_{k} )$. The above procedure is repeated to get r trajectories. Hence, the total numbers of simulations required to determine the sensitivity of the parameters are $r*\left( {k + 1} \right)$ samples^17,37^. Here, we have used $r = 10$, similar to Zhang et al.^37^, and hence the total number of simulations to be performed is 180 (i.e., $10*\left( {17 + 1} \right)$. Furthermore, $\mu_{i}$ is defined as the mean of $\left| {E_{i} } \right|$, and ${\upsigma }_{i}$ as a standard deviation of $E_{i}$.

$$ \mu_{i} = \mathop \sum \limits_{t = 1}^{t = r} \frac{{\left| {E_{i} \left( t \right)} \right|}}{r}; \quad \sigma_{i} = \mathop \sum \limits_{t = 1}^{t = r} \sqrt {\frac{{\left( {E_{i} \left( t \right) - \mu_{i} } \right)^{2} }}{r}} $$

Parameters with a large value of $\mu_{i}$ and $\sigma_{i}$ are considered to be most sensitive with large main and interactive effects. Once $\mu_{i}$ and $\sigma_{i}$ are computed for each parameter, the relative importance (RI) measure of a given parameter is calculated by combining $\mu_{i}$ and $\sigma_{i}$ effects in a relationship by $\beta_{i} = \frac{{\sqrt {\mu_{i}^{2} + \sigma_{i}^{2} } }}{s}$, with equal weightage to $\mu_{i}$ and $\sigma_{i}$, where ‘$s$’ is the standard deviation of $\sqrt {\mu_{i}^{2} + \sigma_{i}^{2} }$ values of different parameters (here ‘*i*’ corresponds to a particular parameter), and is used to normalize the importance of different model parameters^60^.

To perform the Morris sensitivity analysis, we use a newly developed software package by Tong^88^. It is known as the non-intrusive Problem-Solving environment for Uncertainty Analysis and Design Exploration (PSUADE). This software package has a variety of tools to conduct global SA, uncertainty analysis, and parameter calibration with a large set of uncertain parameters. PSUADE has a variety of (~ 15) sampling methods, including the Morris sampling technique, and provides users with the option to choose any method from ~ 10 in-built global SA techniques (where MOAT GSA is one of them). This software package for GSA and uncertainty quantification has been widely used since its development^89–92^.

## Supplementary information

Supplementary Information.

## Data Availability

All the data used in this study is in the public domain and can be downloaded freely.
