# Supplementary material for: Uncertainty quantification based cloud parameterization sensitivity analysis in the NCAR community atmosphere model
Source: Sci Rep. 2020 Oct 15;10:17499. doi: 10.1038/s41598-020-74441-x (PMC7567854; doi:10.1038/s41598-020-74441-x)
Supplement: Supplementary file 1 — Supplementary Information. [file 41598_2020_74441_MOESM1_ESM.docx]

**Supplementary Material**

Uncertainty Quantification Based Cloud Parameterization Sensitivity Analysis in the NCAR Community Atmosphere Model

Raju Pathak^1^, Sandeep Sahany^1,2^, Saroj K. Mishra^1^

^1^Centre for Atmospheric Sciences, Indian Institute of Technology Delhi

^2^Centre for Climate Research, Singapore

Corresponding Author

Sandeep Sahany

---------------------------

Centre for Atmospheric Sciences

Indian Institute of Technology Delhi

Hauz Khas, New Delhi, INDIA

Email: ssahany@cas.iitd.ac.in


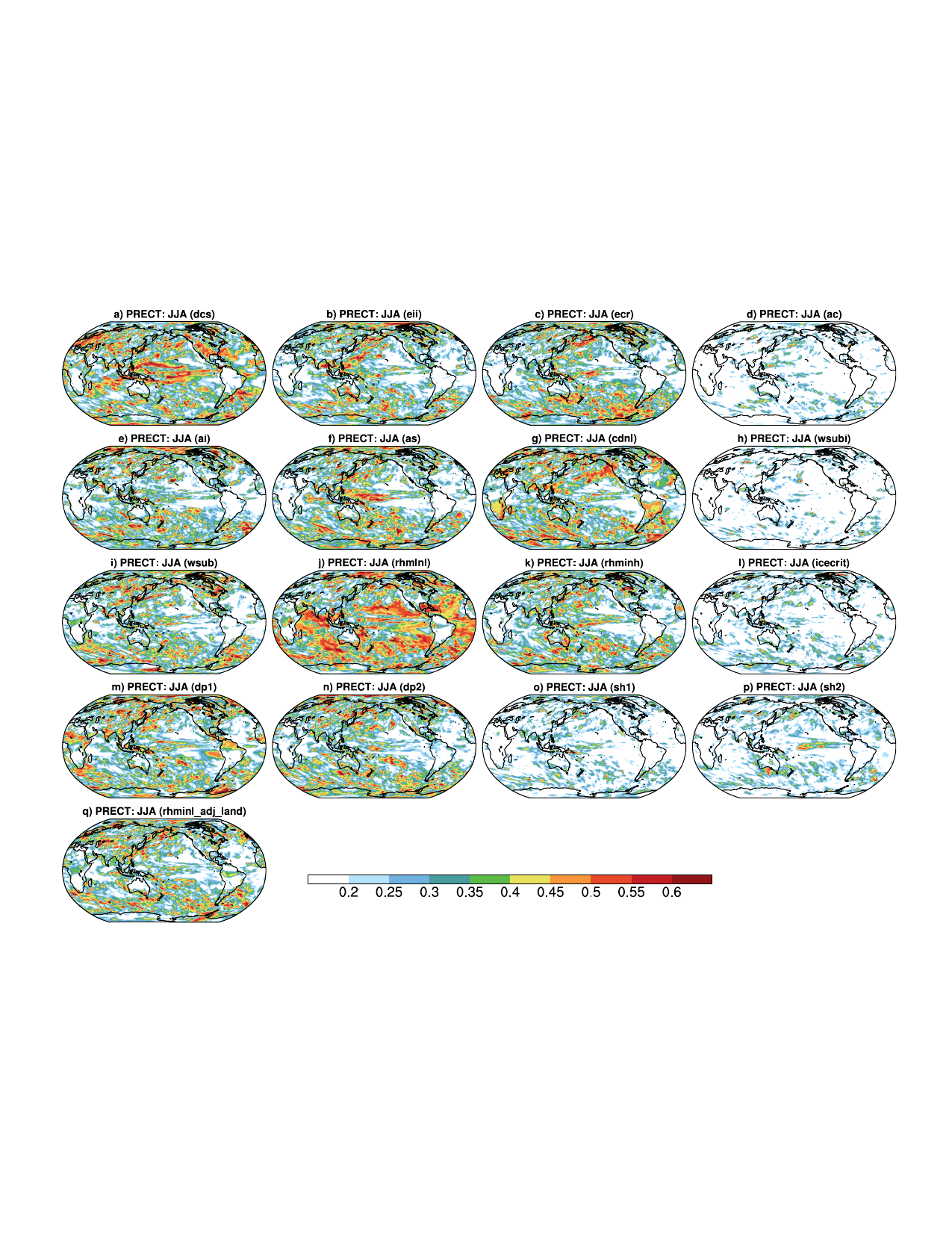


**Figure S1**. Spatial distributions of relative importance (RI) measure for June-August (JJA) mean total precipitation simulation from 180 model simulations for the 17 parameters used for sensitivity analysis. PSUADE version-1.7.8b software (<https://computing.llnl.gov/projects/psuade-uncertainty-quantification>) is used for RI computation using the Morris method at each grid point and NCAR Command Language (NCL) version-6.4.0 (<https://www.ncl.ucar.edu/>) is used for plotting over the global region.


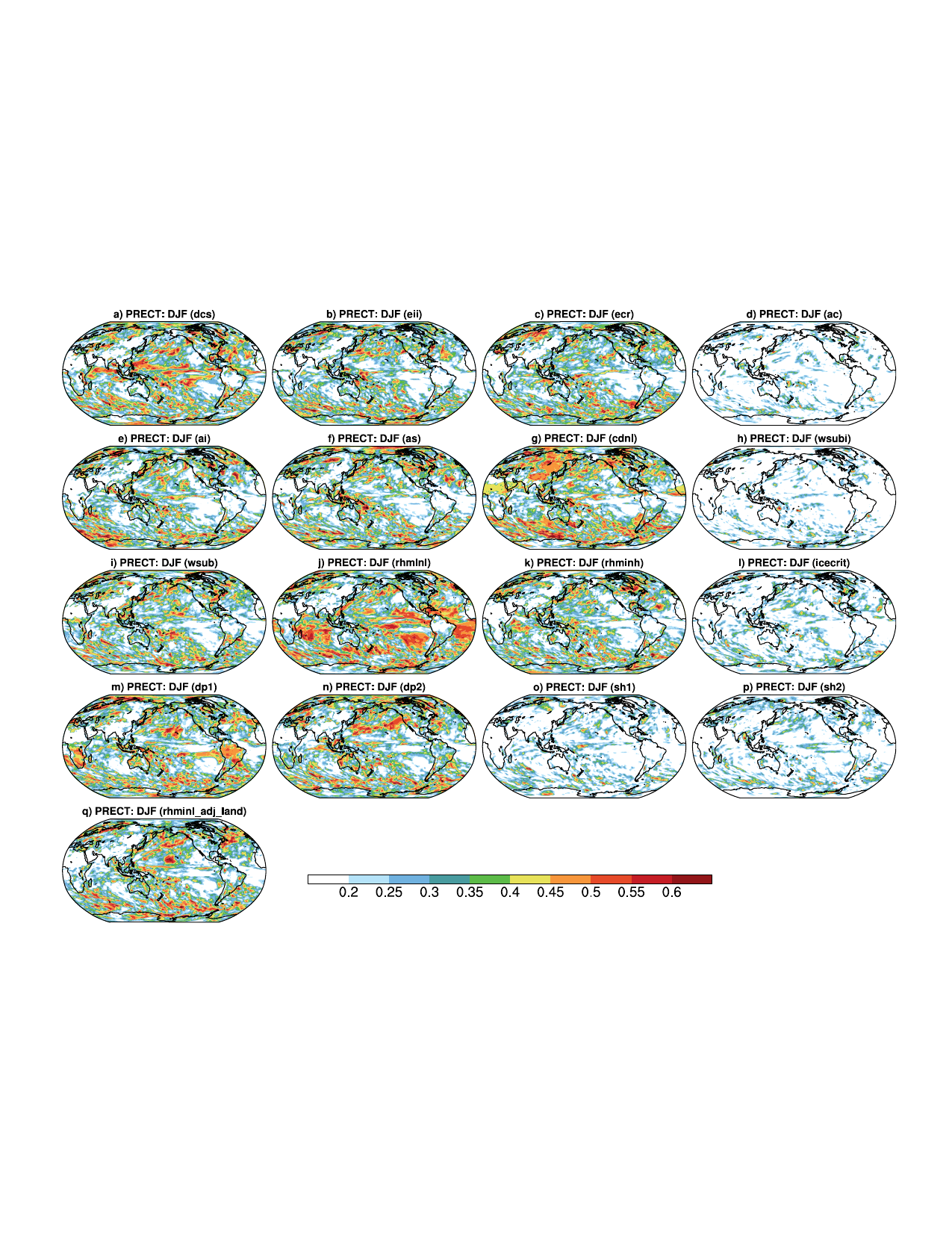


**Figure S2**. Spatial distributions of relative importance (RI) measure for December-February (DJF) mean total precipitation simulation from 180 model simulations for the 17 parameters used for sensitivity analysis. PSUADE version-1.7.8b software (<https://computing.llnl.gov/projects/psuade-uncertainty-quantification>) is used for RI computation using the Morris method at each grid point and NCAR Command Language (NCL) version-6.4.0 (<https://www.ncl.ucar.edu/>) is used for plotting over the global region.


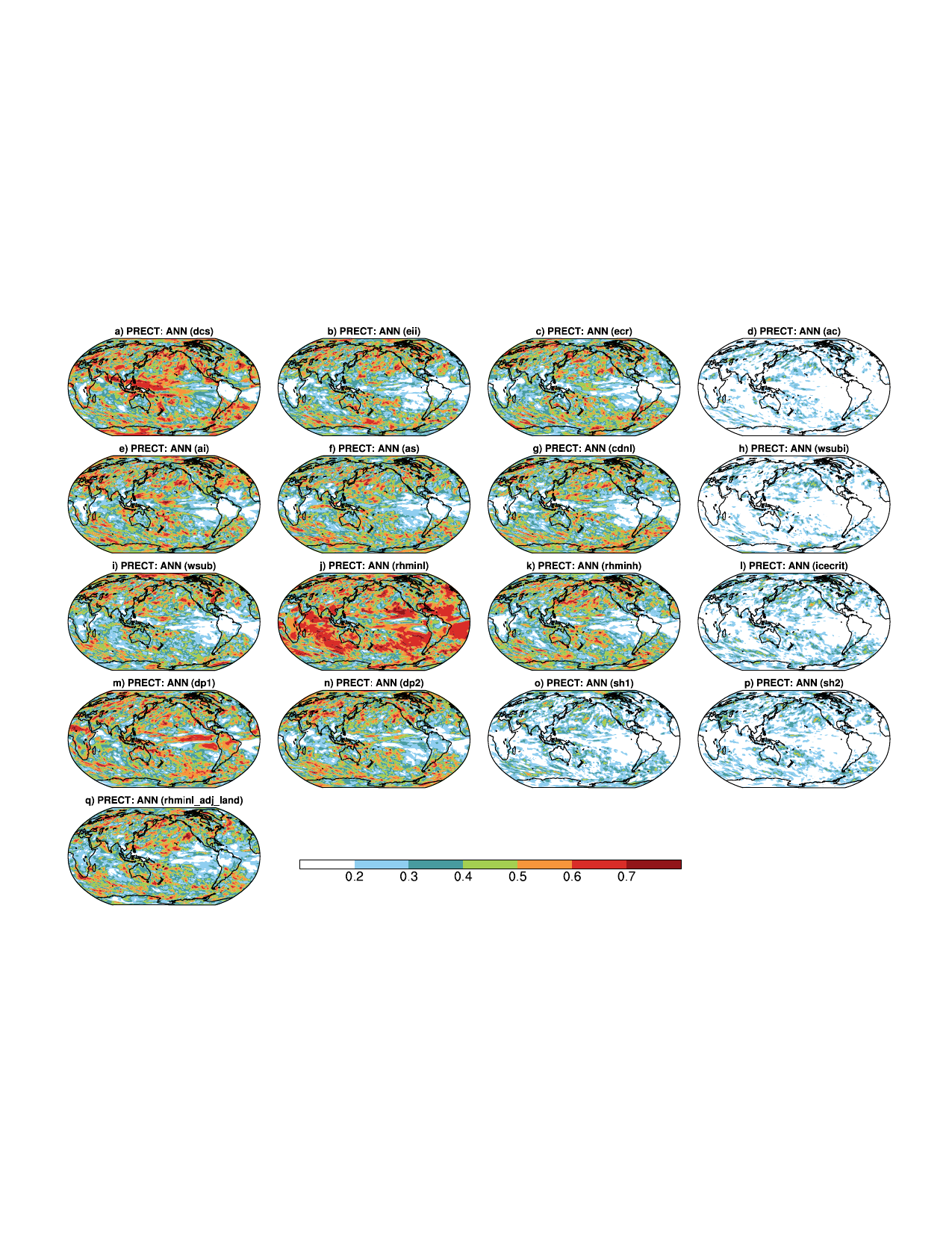


**Figure S3**. Spatial distributions of the normalized main effect (first-order effect) for annual mean precipitation from 180 model simulations for the 17 parameters used for sensitivity analysis. Normalization of the main effect shown in the figure is done by dividing the actual main effect by the global maximum (including the main effects of all parameters). PSUADE version-1.7.8b software (<https://computing.llnl.gov/projects/psuade-uncertainty-quantification>) is used for the first-order effect computation using the Morris method at each grid point, and NCAR Command Language (NCL) version-6.4.0 (<https://www.ncl.ucar.edu/>) is used for plotting over the global region.


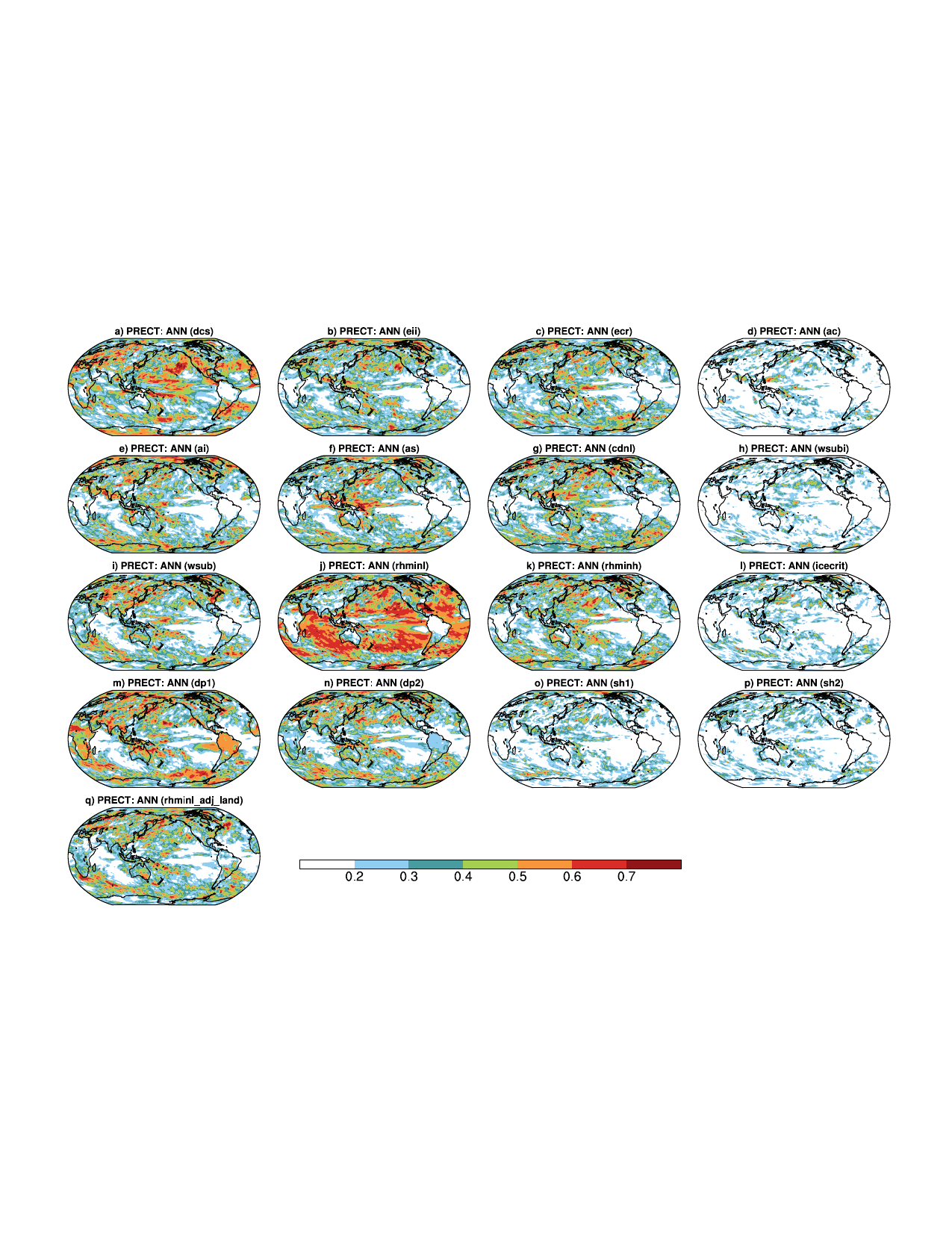


**Figure S4**. Spatial distributions of the importance of normalized interactive effect among the various parameters (higher-order effects) for annual mean precipitation simulation from 180 model simulations for the 17 parameters used for sensitivity analysis. Normalization of the interactive effect shown in the figure is done by dividing the actual interactive effect by the global maximum (including interactive effects of all parameters). PSUADE version-1.7.8b software (<https://computing.llnl.gov/projects/psuade-uncertainty-quantification>) is used for the interactive effect computation using the Morris method at each grid point, and NCAR Command Language (NCL) version-6.4.0 (<https://www.ncl.ucar.edu/>) is used for plotting over the global region.


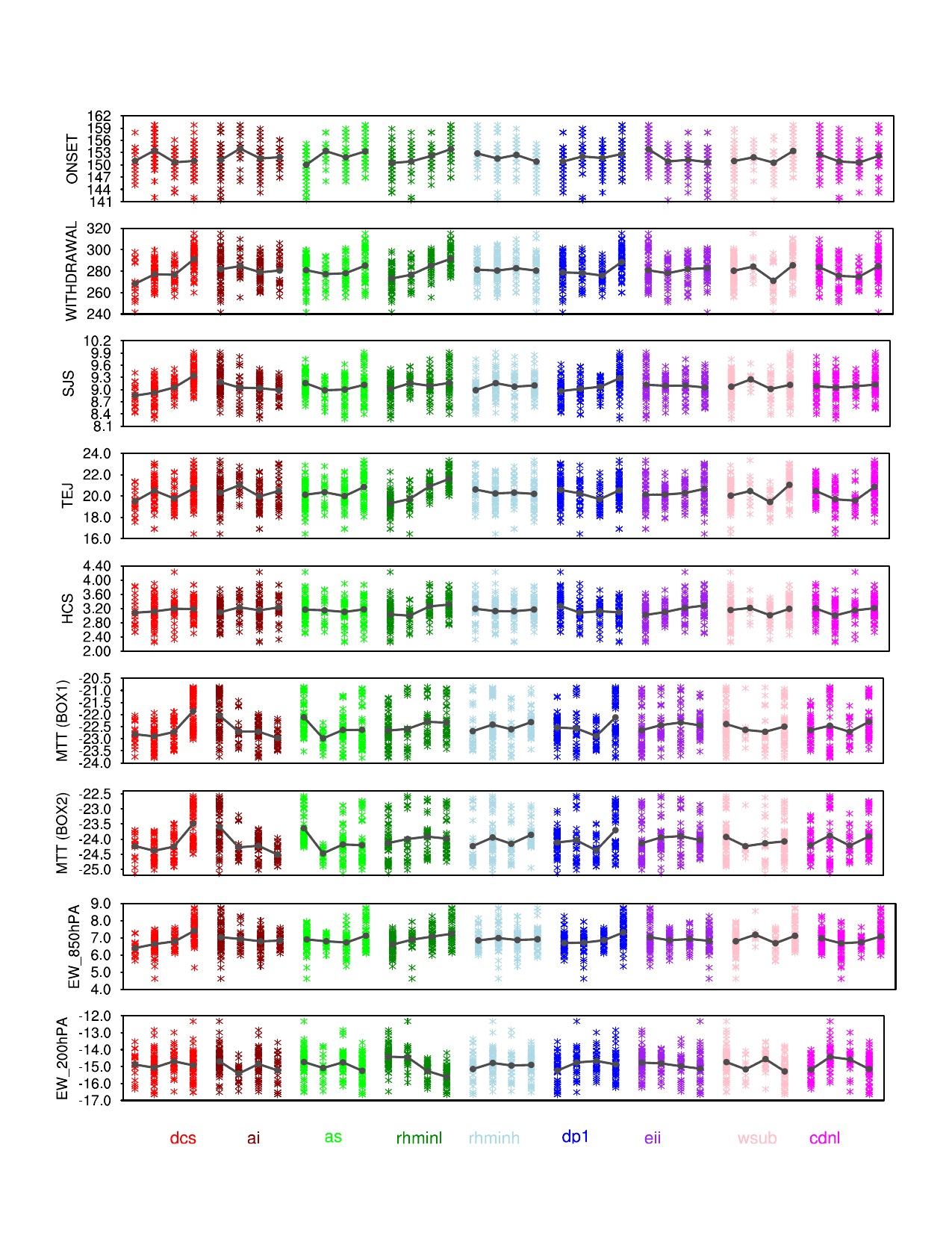


**Figure S5.** The response of vertically averaged meridional tropospheric temperature (MTT) over BOX1 (5°-35°N; 40°-100°E) and BOX2 (5°N-15°S; 40°-100°E), and the zonal wind at 850 hPa (EW_850hPa) and 200 hPa (EW_200hPa) over 0°-15°N; 50°-90°E. A solid dark gray line shows the average effect of a parameter.


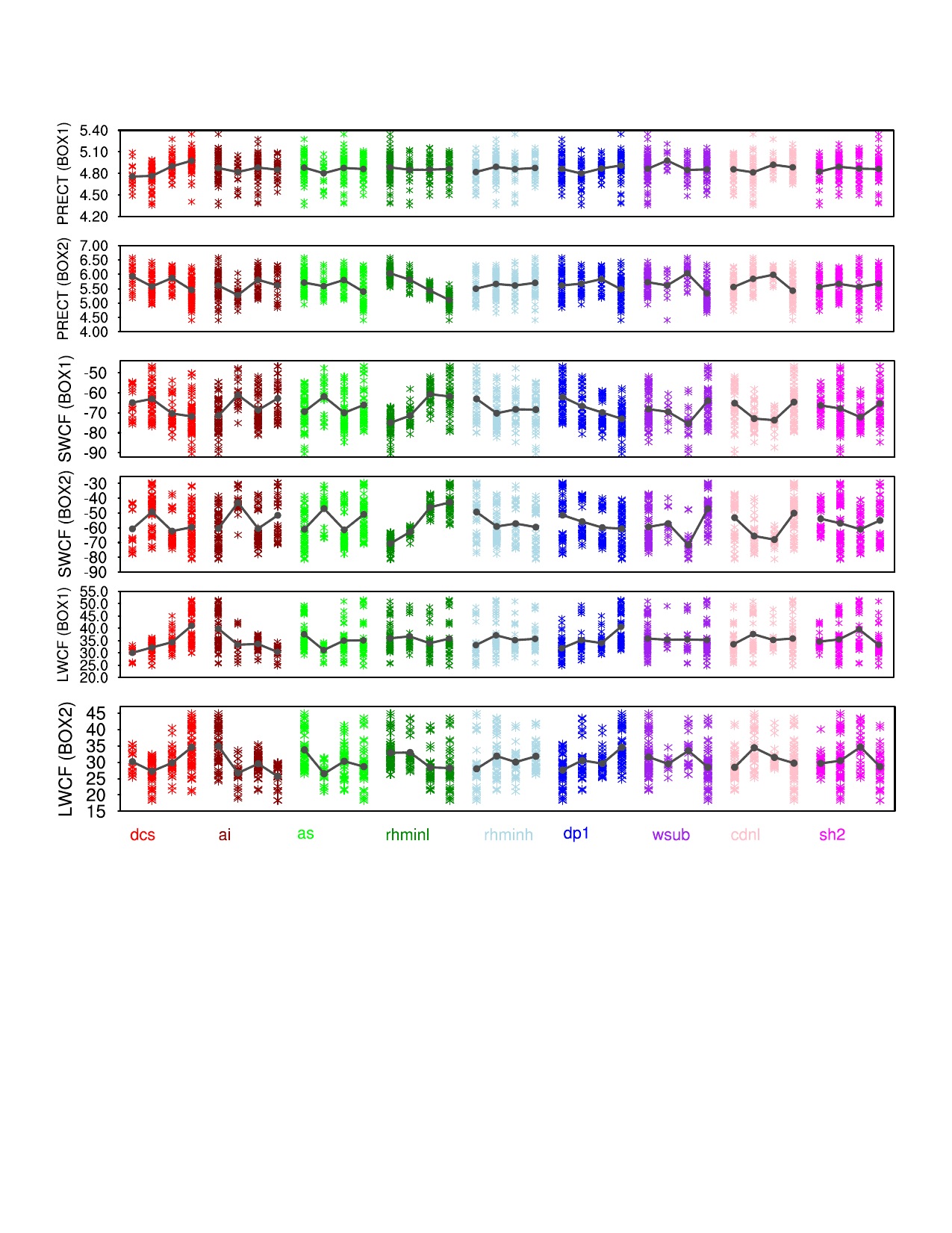


**Figure S6.** The response of PRECT, SWCF, and LWCF over BOX1 (5°-35°N; 40°-100°E) and BOX2 (5°N-15°S; 40°-100°E). A solid dark gray line shows the average effect of a parameter.
